# Association between the triglyceride glucose index and in-hospital and 1-year mortality in patients with chronic kidney disease and coronary artery disease in the intensive care unit

**DOI:** 10.1186/s12933-023-01843-2

**Published:** 2023-05-13

**Authors:** Zixiang Ye, Shuoyan An, Yanxiang Gao, Enmin Xie, Xuecheng Zhao, Ziyu Guo, Yike Li, Nan Shen, Jingang Zheng

**Affiliations:** 1 Department of Cardiology, Peking University China-Japan Friendship School of Clinical Medicine, Beijing, 100029 China; 2 Department of Cardiology, China-Japan Friendship Hospital, 2 Yinghua Dongjie, Chaoyang District, , Beijing, 100029 China; 3 Graduate School of Peking Union Medical College, Chinese Academy of Medical Sciences and Peking Union Medical College, Beijing, 100029 China

**Keywords:** Triglyceride glucose index, In-hospital mortality, Chronic kidney disease, Coronary artery disease, MIMIC-IV database

## Abstract

**Objective:**

This study aimed to explore the association between the triglyceride glucose index (TyG) and the risk of in-hospital and one-year mortality in patients with chronic kidney disease (CKD) and cardiovascular disease (CAD) admitted to the intensive care unit (ICU).

**Methods:**

The data for the study were taken from the Medical Information Mart for Intensive Care-IV database which contained over 50,000 ICU admissions from 2008 to 2019. The Boruta algorithm was used for feature selection. The study used univariable and multivariable logistic regression analysis, Cox regression analysis, and 3-knotted multivariate restricted cubic spline regression to evaluate the association between the TyG index and mortality risk.

**Results:**

After applying inclusion and exclusion criteria, 639 CKD patients with CAD were included in the study with a median TyG index of 9.1 [8.6,9.5]. The TyG index was nonlinearly associated with in-hospital and one-year mortality risk in populations within the specified range.

**Conclusion:**

This study shows that TyG is a predictor of one-year mortality and in-hospital mortality in ICU patients with CAD and CKD and inform the development of new interventions to improve outcomes. In the high-risk group, TyG might be a valuable tool for risk categorization and management. Further research is required to confirm these results and identify the mechanisms behind the link between TyG and mortality in CAD and CKD patients.

**Supplementary Information:**

The online version contains supplementary material available at 10.1186/s12933-023-01843-2.

## Introduction

Cardiovascular disease (CVD) is the primary cause of death and poor health among patients with chronic kidney disease (CKD) [1]. Due to the elevated risks of coronary calcification, small vessel disease, diabetes, hemorrhage, and procedural complications following treatment, CAD patients with CKD appear to be at a higher risk of mortality than CAD patients without CKD [2].

The triglyceride glucose (TyG) index is regarded as a new surrogate measure of insulin resistance and is more diagnostic and predictive of diabetes than blood glucose [3]. Recent researchers have pointed to its association with the development of cardiovascular disease [4], the risk of myocardial infarction [5], in-stent stenosis [6], the severity of coronary artery disease [7], etc. Disturbances in lipid metabolism are common in patients with CKD [8], and studies have suggested a link between TyG and CKD prognosis [9]. The pathophysiology of patients with CKD combined with CAD is complex and the association between the TyG index and mortality in patients with both CKD and CVD has not been explored.

This study aimed to explore the association between the TyG index and the risk of in-hospital and one-year mortality in patients with CKD combined with CAD who were admitted to the intensive care unit (ICU). The results of this study may help in the development of new strategies to enhance patient outcomes in this population and offer important new insights into the function of TyG in predicting patient outcomes.

## Methods

### Data sources and extraction

Medical Information Mart for Intensive Care-IV (MIMIC-IV) is a free, publicly accessible database that includes more than 50,000 ICU admissions from Beth Israel Deaconess Medical Center between 2008 and 2019. (Boston, Massachusetts) [10]. Demographics, vital signs, test findings, and diagnoses using International Classification of Diseases and Ninth Revision (ICD-9) and International Classification of Diseases and Tenth Revision (ICD-10) codes were all included in the MIMIC-IV database. To access these databases, one author (ASY) received the necessary certification, and then retrieved the variables required for the investigation (certification number: 39674606). Individual patient permission was not necessary since patients were not identifiable by their health information in these databases.

This research covered all individuals with CAD and CKD diagnosed via ICD-9 and ICD-10 (https://icd.who.int/browse10/2019/en). CAD was defined according to the codes 410–411 (ICD-9) and I20-I21 (ICD-10), and CKD was defined based on the codes 585, V451 (ICD-9) and N18, Z99.2 (ICD-10). A patient was eliminated if they spent less than six hours in the intensive care unit, were under the age of 18, lacked baseline creatinine readings, or had greater than 30% missing data. Only the initial admission was taken into account if a patient had multiple admissions. Through the use of pgAdmin PostgreSQL tools, data from the MIMIC-IV database were extracted on demographics, lab results, hourly vital signs, comorbidities, medications, surgical procedures, ICU stay specifics, in-hospital mortality, and one-year mortality (version 1.22.1). The "mice" package in R was used to impute the MIMIC-missing IV data [11].

### Feature selection

Following the determination of the relevance of the study variables, feature selection was a crucial step in reducing the number of features in the large dataset. A crucial method of feature selection was the Boruta algorithm, which was based on the random forest classifier method. To compare the Z-score between the genuine features and the shadow features generated by the random forest classifier in each iteration of the model development, Boruta created a duplicate of the features from the original dataset called shadow features. If a feature's Z-score was higher than the highest possible Z-score for shadow features, it was deemed essential and kept; otherwise, it was eliminated [12].

### Statistical analysis

Depending on whether they survived during the hospital stay and lived long enough to be discharged, patients were split into two groups. Fisher's exact probability approach (or the chi-square tests) was used to compare categorical variables that were summarized as percentage-based figures. Continuous variables are expressed as interquartile ranges and the median was tested using the Wilcoxon rank sum test.

The TyG index was computed according to the triglyceride (TG) and fasting blood glucose (FBG) concentrations according to the equation: ln [TG (mg/dl) × FBG (mg/dl)/2]. To evaluate the relationship between the TyG index and the risk of in-hospital mortality and one-year mortality, univariable and multivariable logistic regression analyses, and Cox regression analyses were conducted. Model 1 contained only the TyG index without any other adjustments. In Model 2, gender and age were modified. Model 3 was a completely adjusted model that took feature selection results and clinical experience adjustments into account. Additionally, 3-knotted multivariate restricted cubic spline (RCS) regression was used to assess any potential nonlinear relationships between the TyG index and one-year mortality and in-hospital mortality. Age, sex, estimated glomerular filtration rate (eGFR), diabetes, dialysis, and acute coronary syndrome (ACS) conditions were all taken into account in subgroup analyses, and *P* for interaction was evaluated.

SPSS (26.0, IBM) and R (version 4.1.3, Austria) were used to conduct all statistical analyses and to carry out the work on the Boruta algorithms. A two-sides *P* value of less than 0.05 was considered to indicate statistical significance.

## Results

### Baseline characteristics

**Fig. 1:**
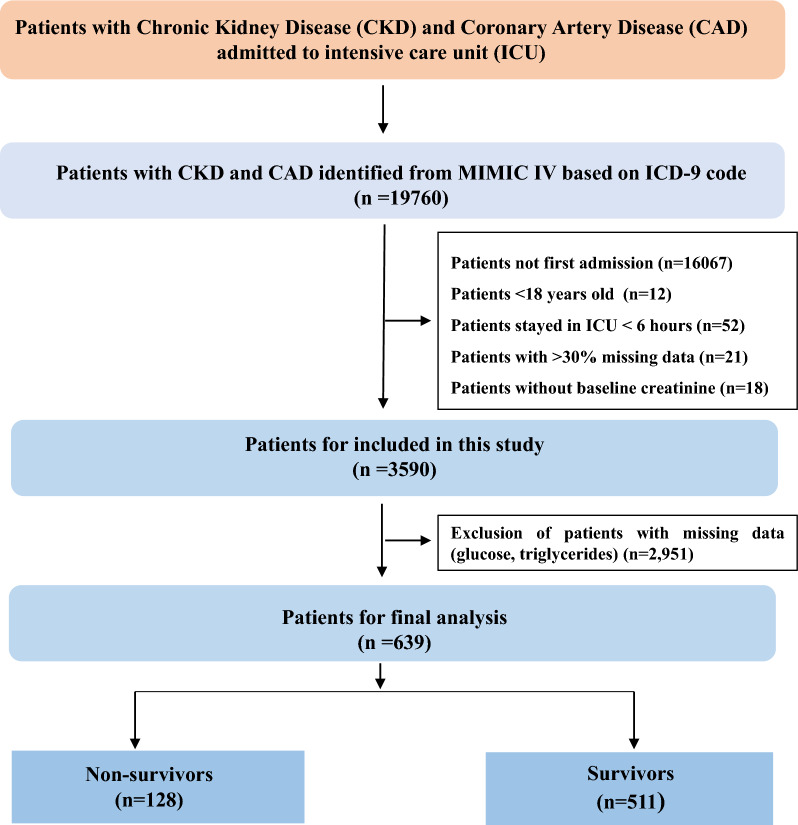
Flowchart illustrating the selection of patients from the MIMIC-IV database. *MIMIC* Medical Information Mart for Intensive Care

The inclusion and exclusion criteria led to the inclusion of 639 CKD patients with CAD from MIMIC-IV in the research (Fig. 1). The median TyG index was 9.1 (8.6, 9.5). Of the 639 CKD patients with CAD who were hospitalized, 102 (15.5%) passed away while 557 others survived. The variations in baseline characteristics are summarized in Table 1 and Additional file 2: Table S1. Serum glucose and TyG index values were greater in patients who passed away while receiving hospital care, and they also had increased risks for liver disease and dialysis (P < 0.001). A total of 128 (19.4%) of the 639 CKD patients with CAD died during the follow-up period (Additional file 2: Table S2).

**Table 1:** The main baseline characteristic for patients included in the study divided by in-hospital situation

	Overall	Survivor	Non-survivor	*P* value
n	639	537	102	
Age (years)	75.0 [66.0,83.0]	74.0 [65.0,83.0]	75.0 [67.0,84.0]	0.804
Male, n (%)	448 (70.1)	370 (68.9)	78 (76.5)	0.158
scr_baseline (mg/dL)	1.3 [1.0,1.9]	1.3 [1.0,1.9]	1.3 [1.0,2.0]	0.993
eGFR (mL/min/1.73m^2^)	53.0 [33.9,73.8]	53.3 [34.2,73.8]	52.7 [32.1,73.8]	0.988
Troponin_mean (ng/mL)	0.4 [0.1,1.1]	0.3 [0.1,1.1]	0.5 [0.1,1.0]	0.059
WBC_mean (K/Ul)	10.2 [8.1,12.5]	9.7 [7.9,11.9]	12.5 [10.6,16.2]	< 0.001
RBC_mean (m/Ul)	3.2 [2.9,3.7]	3.3 [2.9,3.7]	3.1 [2.7,3.4]	0.001
Hemoglobin_mean (g/dL)	9.7 [8.7,10.8]	9.8 [8.8,11.0]	9.1 [8.3,10.0]	< 0.001
Hematocrit_mean (%)	29.4 [26.8,33.2]	29.8 [27.0,33.5]	28.1 [26.1,30.7]	0.001
platelet_mean (K/uL)	203.0 [153.7,260.6]	207.0 [160.8,262.8]	177.4 [127.2,247.5]	0.002
ALT_mean (IU/L)	24.8 [16.0,49.0]	22.5 [14.8,41.7]	39.5 [20.5,115.2]	< 0.001
Bilirubin_total_mean (mg/dL)	0.6 [0.4,1.0]	0.6 [0.3,0.8]	1.0 [0.5,1.9]	< 0.001
Creatinine_mean (mg/dL)	1.9 [1.4,2.8]	1.8 [1.3,2.6]	2.3 [1.7,3.5]	< 0.001
Sodium_mean (mEq/L)	138.6 [136.2,141.0]	138.7 [136.5,141.0]	137.7 [134.8,140.9]	0.059
Total_calcium_mean (mg/dL)	8.6 [8.3,9.0]	8.6 [8.3,8.9]	8.6 [8.1,9.0]	0.48
Free_calcium_mean (mmol/L)	1.1 [1.1,1.2]	1.1 [1.1,1.2]	1.1 [1.1,1.1]	0.006
Inr_mean	1.3 [1.1,1.5]	1.2 [1.1,1.4]	1.5 [1.3,1.8]	< 0.001
PT_mean (seconds)	14.0 [12.6,16.4]	13.8 [12.4,15.8]	16.2 [13.8,20.1]	< 0.001
Triglycerides_mean (mg/dl)	121.3 [88.0,174.1]	123.0 [88.0,173.0]	111.5 [83.5,178.2]	0.665
Cholesterol_mean (mg/dl)	137.0 [108.0,168.0]	137.0 [111.0,167.5]	127.0 [99.0,180.8]	0.265
LDL_mean (mg/dl)	64.0 [42.0,98.0]	66.0 [44.0,98.0]	56.5 [32.8,101.8]	0.243
HDL_mean (mg/dl)	39.0 [29.0,50.5]	40.0 [29.0,51.0]	34.0 [25.0,49.5]	0.018
Glucose_mean (mg/dL)	136.8 [114.2,169.6]	131.8 [112.2,163.2]	161.4 [132.0,197.7]	< 0.001
TYG index	9.1 [8.6,9.5]	9.0 [8.6,9.4]	9.2 [8.7,9.7]	0.012
SOFA	6.0 [3.0,9.0]	5.0 [3.0,8.0]	9.0 [6.0,11.0]	< 0.001
HR_mean (beats/minute)	79.7 [71.5,89.4]	78.8 [71.2,87.6]	88.6 [78.4,95.7]	< 0.001
SpO_2__mean	96.8 [95.6,97.8]	96.7 [95.6,97.8]	97.1 [95.7,98.1]	0.13
Aspirin, n (%)	555 (86.9)	472 (87.9)	83 (81.4)	0.104
Statin, n (%)	538 (84.2)	465 (86.6)	73 (71.6)	< 0.001
Warfarin, n (%)	165 (25.8)	146 (27.2)	19 (18.6)	0.091
Diabetes, n (%)	357 (55.9)	293 (54.6)	64 (62.7)	0.156
HT, n (%)	587 (91.9)	491 (91.4)	96 (94.1)	0.477
ACS, n (%)	236 (36.9)	206 (38.4)	30 (29.4)	0.109
Dialysis, n (%)	149 (23.3)	101 (18.8)	48 (47.1)	< 0.001

*TyG* triglyceride glucose; *scr* serum creatinine; *eGFR* estimated glomerular filtration rate; *ACS* acute coronary syndrome; *HT* hypertension; *max* maximum; min, minimum; *WBC* white blood cell; *RBC* red blood cell; *ALT* alanine aminotransferase; *INR* International Normalized Ratio; *PT* prothrombin time; *SOFA* sequential organ failure assessment; *HR* heart rate; *SpO*_*2*_ oxyhemoglobin saturation

### Feature selection

**Fig. 2:**
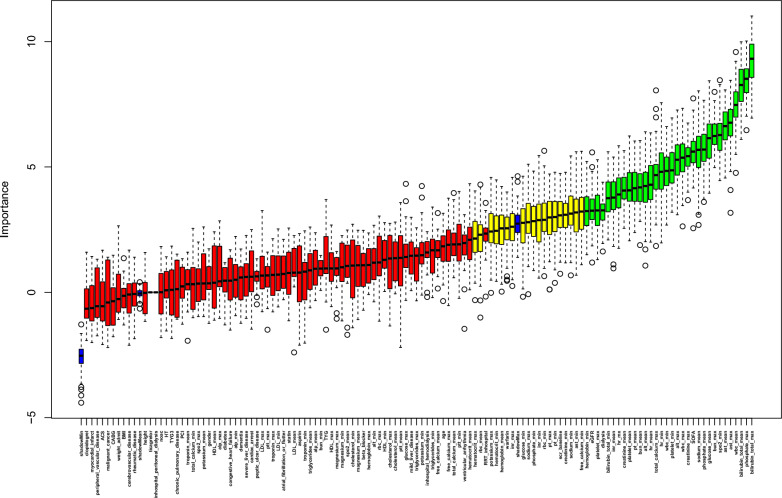
Feature selection for the relationship between various TyG indices and in-hospital mortality analyzed by the Boruta algorithm. The horizontal axis shows the name of each variable, while the vertical axis represents the Z-value of each variable. The box plot depicts the Z-value of each variable in the model calculation, with green boxes representing important variables, yellow representing tentative attributes, and red representing unimportant variables. *scr* serum creatinine, *eGFR* estimated glomerular filtration rate, ACS acute coronary syndrome, *HT*, hypertension; *max*, maximum; *min*, minimum, *WBC* white blood cell; RBC, red blood cell, *ALT* alanine aminotransferase, *INR* International Normalized Ratio, *PT* prothrombin time, *SOFA* sequential organ failure assessment, *HR* heart rate, *SpO*_*2*_ oxyhemoglobin saturation

Thirty-two and 34 variables that were the most associated with in-hospital and one-year mortality, respectively, were confirmed using the Boruta method, (Fig. 2, Additional file 1: Fig. S1, and Additional file 2: Tables S3, S4). Although several important characteristics, such as ACS, dialysis, diabetes and hypertension and medication situation, such as aspirin and statin use, were disregarded due to the low Z-value in comparison to the shadow feature, they were nonetheless included in the analysis based on prior research and clinical experience. Factors were chosen for the final complete adjustment model when in the Boruta analysis, their Z-scores were higher than the shadow features or when added to the model, they had the largest matched effect (odds ratio or hazard ratio) among a group of biomarkers (max, mean and min) or they were based on previous findings and clinical constraints.

### TyG index and in-hospital mortality relationship

**Fig. 3:**
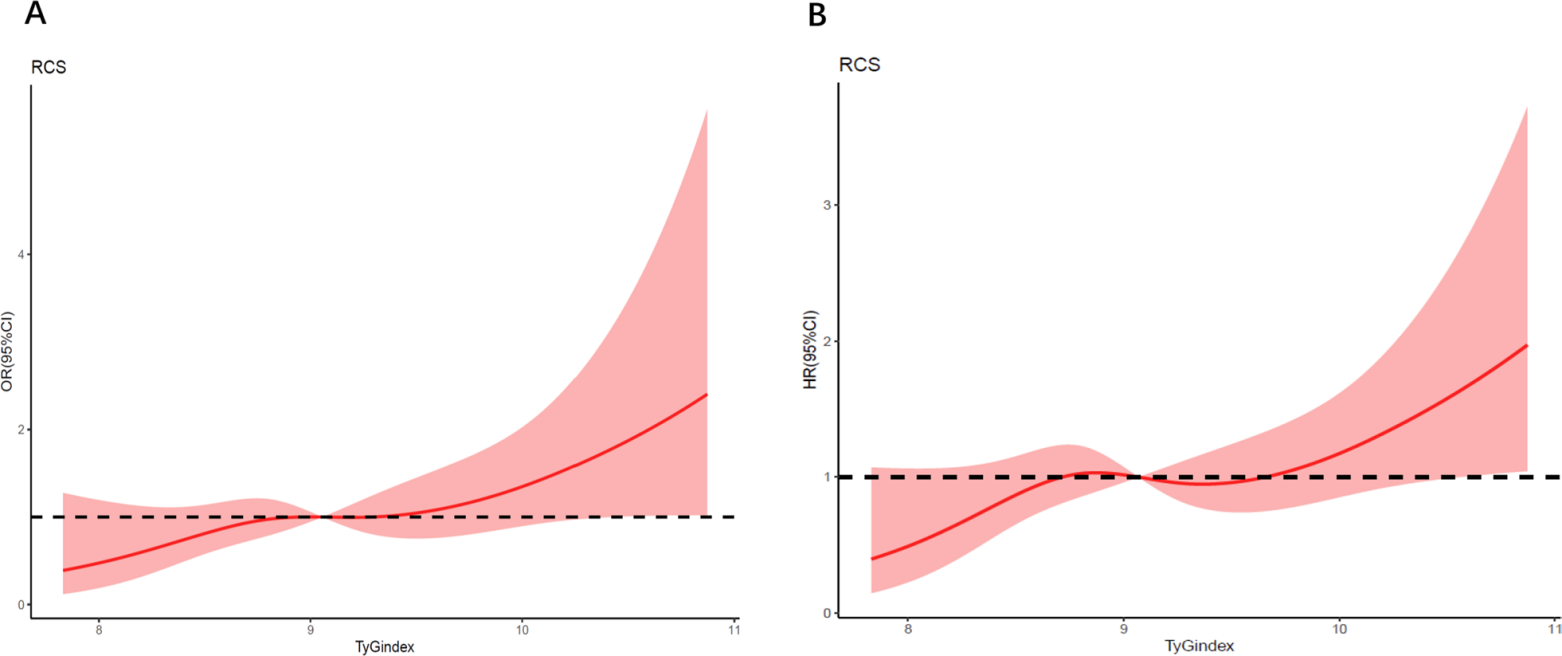
Multivariable RCS regression showed the nonlinear association between the TyG index and in-hospital (**A**) and one-year (**B**) mortality after full adjustment. The cutoff values in the plot of in-hospital death risk were 8.869 and 9.373, while the cutoff values for one-year mortality were 8.716, 9.037, and 9.664. TyG, triglyceride glucose; RCS, restricted cubic spline

According to the database, there were 102 in-hospital deaths out of 639 patients (16.0%). The TyG index was found to have a nonlinear relationship with the probability of dying in the hospital according to the multivariable RCS model. When the TyG index was between 8.869 and 9.373, a plateau phase was noticed, and the hazard ratio (HR) value of the TyG index was near 1. The risk of in-hospital death increased with the TyG index, whether it was less than 8.869 or larger than 9.373 (Fig. 3A). The risk of in-hospital mortality and the TyG index were not yet significantly associated in this range.

We defined three categories of patients based on the TyG index: Q1 (TyG ≤ 8.869), Q2 (8.869 < TyG ≤ 9.373), and Q3 (TyG > 9.373). The results of multivariable logistic regression (Table 2, Model 3) showed that the TyG index increased the probability of dying in the hospital (OR 1.678, 95% confidence interval [CI] 1.113–2.531) after adjusting for all impact factors identified by Boruta analysis and clinical experience. Compared to the lowest TyG index in Q1 (Table 2, *P* for trend 0.026), the OR for the incidence of in-hospital death decreased in Q2 (OR 0.968, 95% CI 0.487–1.925); however, it climbed in Q3 (OR 2.165, 95% CI 1.122–4.179).

**Table 2:** The association between various TyG index groups and in-hospital mortality

	Model1	Model2	Model3
TyG index	1.559 (1.145–2.123)	1.611 (1.171–2.215)	1.678 (1.113–2.531)
TyG			
Q1	ref	ref	ref
Q2	0.738 (0.420–1.298)	0.759 (0.430–1.343)	0.968 (0.487–1.925)
Q3	1.708 (1.049–2.779)	1.754 (1.054–2.876)	2.165 (1.122–4.179)
*P* for trend	0.008	0.009	0.026
Model2	Adjusted for age, sex		
Model3	Adjusted for age, sex, ACS, dialysis, HT, SOFA, eGFR, RBC_mean, hematocrit_min, platelet_mean, ALT_mean, bilirubin_total_mean, creatinine_mean, potassium_max, sodium_mean, total_calcium_max, free_calcium_min, phosphate_min, INR_mean, PT_mean, HR_mean, SpO_2__min, statin, aspirin, warfarin		

*TyG* triglyceride glucose; *eGFR* estimated glomerular filtration rate; *ACS* acute coronary syndrome; *HT* hypertension; *max* maximum; min, minimum; *RBC* red blood cell; *ALT* alanine aminotransferase; *INR* International Normalized Ratio; *PT* prothrombin time; *PTT* partial thromboplastin time; *SOFA* sequential organ failure assessment; *HR* heart rate; *SpO*_*2*_ oxyhemoglobin saturation

### One-year mortality and the TyG index correlation

A total of 128 of 639 (20.0%) participants died during the one-year follow-up period. The TyG index was nonlinearly related to the risk of in-hospital death according to the multivariable RCS model. When the TyG index was less than 8.716 or more than 9.664, the 1-year risk of death was positively correlated with the value of the TyG index. The HR value was near 1 when the TyG index ranged from 8.716 to 9.664 with a cutoff value of 9.037 (Fig. 3B).

**Fig. 4:**
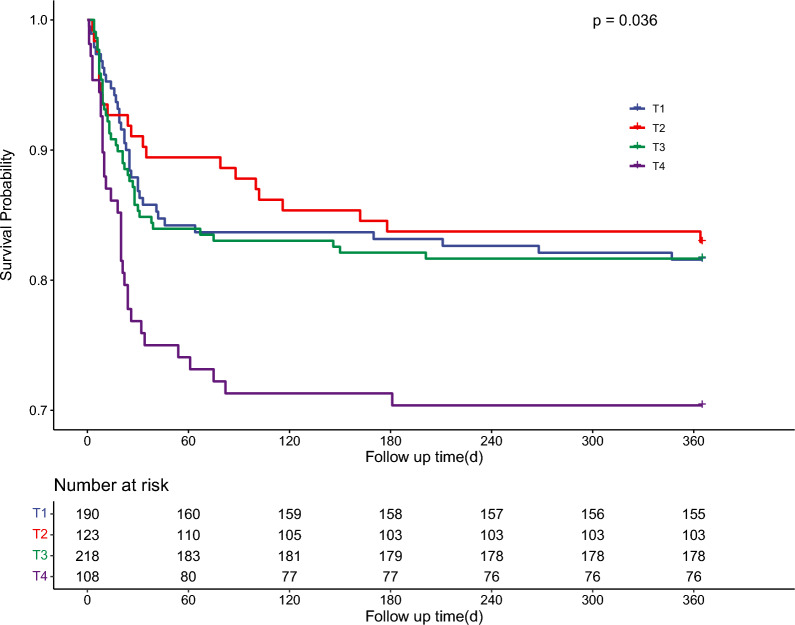
Kaplan–Meier analysis results illustrated the cumulative incidence of one-year mortality in patients with both CKD and CAD in various TyG index groups. *TyG* triglyceride glucose, *CKD* chronic kidney disease, *CAD* coronary artery disease

As a result, the participants were divided into four groups based on various TyG indices: T1 (TyG ≤ 8.716), T2 (8.716 < TyG ≤ 9.037), T3 (9.037 < TyG ≤ 9.664), and T4 (TyG > 9.664). The Kaplan–Meier analysis plot showed a significant difference among various TyG index groups (Fig. 4).

Overall, multivariable Cox regression analysis revealed an association between the TyG index score and an elevated risk of one-year mortality (HR 1.343, 95% CI 1.020–1.769, *P* < 0.05). While the HRs of T2 and T3 were reduced (HR 0.750, 95% CI 0.423–1.331, and HR 0.929, 95% CI 0.568–1.520, respectively), the HR of T4 was increased compared to that of T1 (HR 1.627, 95% CI 1.058–2.766). The trend from T1 to T4 was statistically significant but not very pronounced (Table 3, *P* for trend 0.049).

**Table 3:** The association between various TyG index groups and one-year mortality

	Model1	Model2	Model3
TyG index	1.403 (1.096–1.795)	1.430 (1.109–1.843)	1.343 (1.020–1.769)
TyG			
T1	ref	ref	ref
T2	0.916 (0.533–1.574)	0.934 (0.543–1.606)	0.750 (0.423–1.331)
T3	1.010 (0.641–1.589)	1.032 (0.651–1.635)	0.929 (0.568–1.520)
T4	1.762 (1.091–2.846)	1.825 (1.111–2.998)	1.627 (1.058–2.766)
*P* for trend	0.041	0.038	0.049
Model2	Adjusted for age, sex		
Model3	Adjusted for age, sex, ACS, dialysis, HT, SOFA, eGFR, RBC_mean, hematocrit_max, platelet_mean, ALT_mean, bilirubin_total_mean, creatinine_mean, potassium_max, sodium_mean, total_calcium_max, free_calcium_min, phosphate_min, INR_mean, PT_mean, HR_mean, SpO_2__min, statin, aspirin, warfarin		

*TyG* triglyceride glucose; *eGFR* estimated glomerular filtration rate; *ACS* acute coronary syndrome; *HT* hypertension; *max* maximum; *min* minimum; *RBC* red blood cell; *ALT* alanine aminotransferase; *INR* International Normalized Ratio; *PT* prothrombin time; *PTT*, partial thromboplastin time; *SOFA*, sequential organ failure assessment; *HR*, heart rate; *SpO*_*2*_, oxyhemoglobin saturation

### Subgroup analysis

To confirm the relationship between the TyG index and in-hospital mortality as well as one-year mortality stratified by age, sex, kidney function and health status, subgroup analyses were carried out. Age and eGFR level were found to interact with the relationship between the TyG index and in-hospital deaths, whereas sex interacted with the TyG index and one-year mortality. Elderly patients (≥ 75 years), males, those with higher eGFR levels, non-ACS and non-dialysis participants continued to show a similar association between the TyG index and in-hospital risk. The link between TyG and 1-year mortality risk was the same among male and non-ACS patients (Table 4 and Additional file 2: Table S5).

**Table 4:** The subgroup analysis results of the multivariable-adjusted ORs for the association between the TyG index and hospital mortality

	CASE	TOTAL	Q1	Q2	Q3	*P* for trend	*P* for interaction
AGE, year							
≥ 75	54	320	ref	0.799 (0.314–2.031)	2.961 (1.224–7.166)	0.016	0.028
< 75	48	319	ref	1.040 (0.329–3.290)	1.499 (0.507–4.425)	0.695	
Sex							
Male	78	448	ref	0.996 (0.430–2.306)	2.671 (1.216–5.869)	0.016	0.194
Female	24	191	ref	0.721 (0.531–0.979)	1.406 (1.035–1.910)	0.005	
ACS							
Yes	30	236	ref	1.091 (0.249–4.782)	1.939 (0.413–9.097)	0.647	0.477
No	72	403	ref	0.962 (0.398–2.325)	2.549 (1.123–5.787)	0.030	
eGFR, mL/min/1.73m^2^							
≥ 30	81	506	ref	0.959 (0.419–2.195)	2.507 (1.133–5.547)	0.030	0.023
< 30	31	133	ref	0.789 (0.129–4.724)	1.103 (0.152–8.004)	0.934	
Dialysis							
Yes	48	149	ref	0.594 (0.143–2.467)	1.015 (0.407–2.529)	0.761	0.320
No	54	490	ref	0.857 (0.128–3.374)	2.575 (1.078–6.152)	0.043	

*TyG* triglyceride glucose, *OR* odds ratio, *eGFR* estimated glomerular filtration rate; *ACS* acute coronary syndrome

## Discussion

This study provided evidence that TyG is a predictor of in-hospital and one-year mortality in patients with CAD and CKD in the ICU. Consequently, this study demonstrated that within a certain range, a higher TyG index indicated a higher incidence of in-hospital mortality and 1-year mortality in patients with both CKD and CAD. After adjusting for confounders, the TyG index was still significantly related to increasing in-hospital mortality (OR = 1.678). In addition, the highest TyG index values enhanced mortality risk by 34.3% over the 1-year follow-up.

The TyG index has been widely established as a good and novel surrogate for IR [13] and has proved to be independently linked with the incidence of DM [14]. Based on this, a higher risk of cardiovascular events [15], CKD [16], and death [17] among individuals with an elevated level of the TyG index has also been reported. After a median follow-up of 98.2 months, Liu et al. [18] showed that the TyG index carried a 0.1-fold elevated risk of death in the general population. According to a retrospective observational study [19], the TyG score and ICU mortality among critical stroke patients showed a substantial connection and predictive value. Another study [20] examining the relationship between the TyG index and ICU mortality in critically sick patients also produced similar findings. Nevertheless, there are few data to support relationships between the TyG index and patients' risk of mortality from both CKD and CAD.

After the multivariate regression analysis and the subgroup analysis, we showed that the TyG index has independent relevance to the risk of in-hospital mortality and 1-year mortality among patients with both CKD and CAD. Taking the TyG index as a simple method of evaluating the extent of IR, our findings demonstrated that IR was independently related to mortality. The pathogenesis behind this effect between the TyG index and mortality is not fully understood, and it might be explained by the severity of the disease reflected by IR. IR, characterized by a significant decline in glucose metabolism in response to insulin, is thought to be a contributory factor of chronic hyperglycemia, dyslipidemia, and hypertension [21]. Further oxidative stress and inflammatory responses lead to endothelial dysfunction and cell damage [22] [23].

In the subgroup analysis, our study found that the nonlinear relationship between the TyG index and in-hospital mortality in patients with CAD and CKD in the ICU was consistent with elderly, both male and female, non-ACS, non-dialysis and mild CKD (eGFR > 30 mL/min/1.73m^2^) patients, and had an interaction with age and eGFR (*P* for interaction < 0.05). A higher TyG index in elderly patients with CAD and CKD in the ICU was associated with in-hospital mortality. The reason may be explained by the mechanism that increased visceral obese tissue in older individuals amplifies the effect of TyG on mortality [24]. CKD has a large impact on lipid metabolism and composition, and lipid structure is altered in patients with moderate-to-severe CKD compared with mild CKD patients. Proinflammatory factors in end-stage renal disease (ESRD) patients, through the NF-κB family, activate the ubiquitination pathway to promote lipoprotein catabolism [25]. Statin use improves prognosis in patients with mild to moderate renal failure, but benefit in ESRD patients is inconclusive [1]. We found an interaction between eGFR on TyG index and prognosis in patients with CKD and CAD. The prognostic effect of the TyG index was more pronounced in patients with mild to moderate CKD combined with CAD than in patients with severe CKD combined with CAD, although the confidence interval for the severe CKD and CAD patients crossed 1. Interestingly, we seemingly found a potential positive relationship between the TyG index and both in-hospital and one-year mortality in patients with CKD combined with ACS (although *P* for trend > 0.05). ACS is mostly due to rupture or invasion of coronary atherosclerotic plaques and secondary complete or incomplete occlusive thrombosis. It has been shown that TyG is associated with in-stent restenosis in patients with coronary artery disease, which increases mortality in patients with ACS[6]. In patients with CKD combined with ACS, lipid metabolism is more disturbed and prone to the formation of lipid plaques resulting in patient death[26].

Our findings hold important implications for clinical practice and patient care. In our study, we found that higher TyG was associated with increased in-hospital and one-year mortality in a population of patients with both CAD and CKD within a specific range. This result suggests that TyG may serve as a valuable tool for risk stratification and management in this high-risk patient population. To address the elevated risk posed by high TyG levels, a comprehensive approach to risk management is needed. This approach should include aggressive management of cardiovascular risk factors such as lipid control, blood pressure control, and smoking cessation. Regular monitoring and timely intervention in patients with elevated TyG levels are critical in reducing the incidence of adverse outcomes.

The role of the TyG index in patients with CKD and CAD in the ICU is being examined for the first time in our investigation, and this study highlights the need for a multifaceted approach to risk management in patients with CAD and CKD, with the TyG index serving as a valuable tool in this process. However, our findings are limited by several factors. First, the study's sample size was modest, and the follow-up period was short making it imperative for future studies to focus on larger sample sizes with extended follow-up periods to provide more robust evidence to support our findings. Second, the availability of data in the database utilized for the study may have omitted important clinical information, such as the patient's dialysis situation, which could impact lipid and glucose metabolism. Next, the data collected in the study were only available at baseline, which means that changes in TyG levels during the follow-up period could not be assessed. Last, the public database used in this study was based on patients who were admitted to Beth Israel Deaconess Medical Center; therefore, Berkson's bias, which is a kind of selection bias was inevitable. To overcome these limitations, future studies should aim to capture comprehensive clinical information and track changes in TyG index values over time.

## Conclusions

As a result, our research shows that TyG is a predictor of one-year mortality and in-hospital mortality in ICU patients with CAD and CKD. In this high-risk group, TyG might be a valuable tool for risk categorization and management. Further research is required to confirm these results and identify the mechanisms behind the link between TyG and mortality in CAD and CKD patients.

## Supplementary Information

**Additional file 1: Figure S1** Feature selection for the relationship between various TyG indices and one-year mortality analyzed by the Boruta algorithm.

**Additional file 2: Table S1.** Baseline characteristics for patients included in the study divided by in-hospital survival status. **Table S2** Baseline characteristics for patients included in the study divided by one-year survival status. **Table S3** Boruta algorithm analysis results for feature selection in the relationship between various TyG indices and in-hospital mortality.** Table S4** Boruta algorithm analysis results for feature selection in the relationship between various TyG indices and one-year mortality.

## Data Availability

The data supporting this study’s findings are available from the Medical Information Mart for Intensive Care IV (MIMIC-IV), but restrictions apply to the availability of these data, which were used under license for the current study, and so are not publicly available. Data are, however, available from the author Shuoyan An (anshuoyan@126.com) upon reasonable request and with permission of MIMIC.

## Abbreviations

TyG: Triglyceride glucose
CKD: Chronic kidney disease
CAD: Coronary artery disease
MIMIC-IV: Medical Information Mart for Intensive Care IV
LR: Logistic regression
ESRD: End stage renal disease
ML: Machine learning
ICD-9: International Classification of Diseases and Ninth Revision
ICD-10: International Classification of Diseases and tenth Revision
ICU: Intensive care unit
scr: Serum creatinine
eGFR: Estimated glomerular filtration rate
ACS: Acute coronary syndrome
HT: Hypertension
PCI: Percutaneous coronary intervention
CABG: Coronary artery bypass grafting
NOAC: Non-vitamin K antagonist oral anticoagulant
CRRT: Continuous renal replacement therapy
max: Maximum
min: Minimum
WBC: White blood cell
RBC: Red blood cell
ALT: Alanine aminotransferase
AST: Aspartate aminotransferase
ALP: Alkaline phosphatase
BUN: Blood urea nitrogen
INR: International normalized ratio
PT: Prothrombin time
PTT: Partial thromboplastin time
SOFA: Sequential organ failure assessment
sbp: Systolic blood pressure
dbp: Diastolic blood pressure
mbp: Mean blood pressure
HR: Heart rate
SpO_2_: Oxyhemoglobin saturation
