# Supplementary figures and images for: Association between the triglyceride glucose index and in-hospital and 1-year mortality in patients with chronic kidney disease and coronary artery disease in the intensive care unit
Source: Cardiovasc Diabetol. 2023 May 13;22:110. doi: 10.1186/s12933-023-01843-2 (PMC10183125; doi:10.1186/s12933-023-01843-2)

Importance

0 5 10

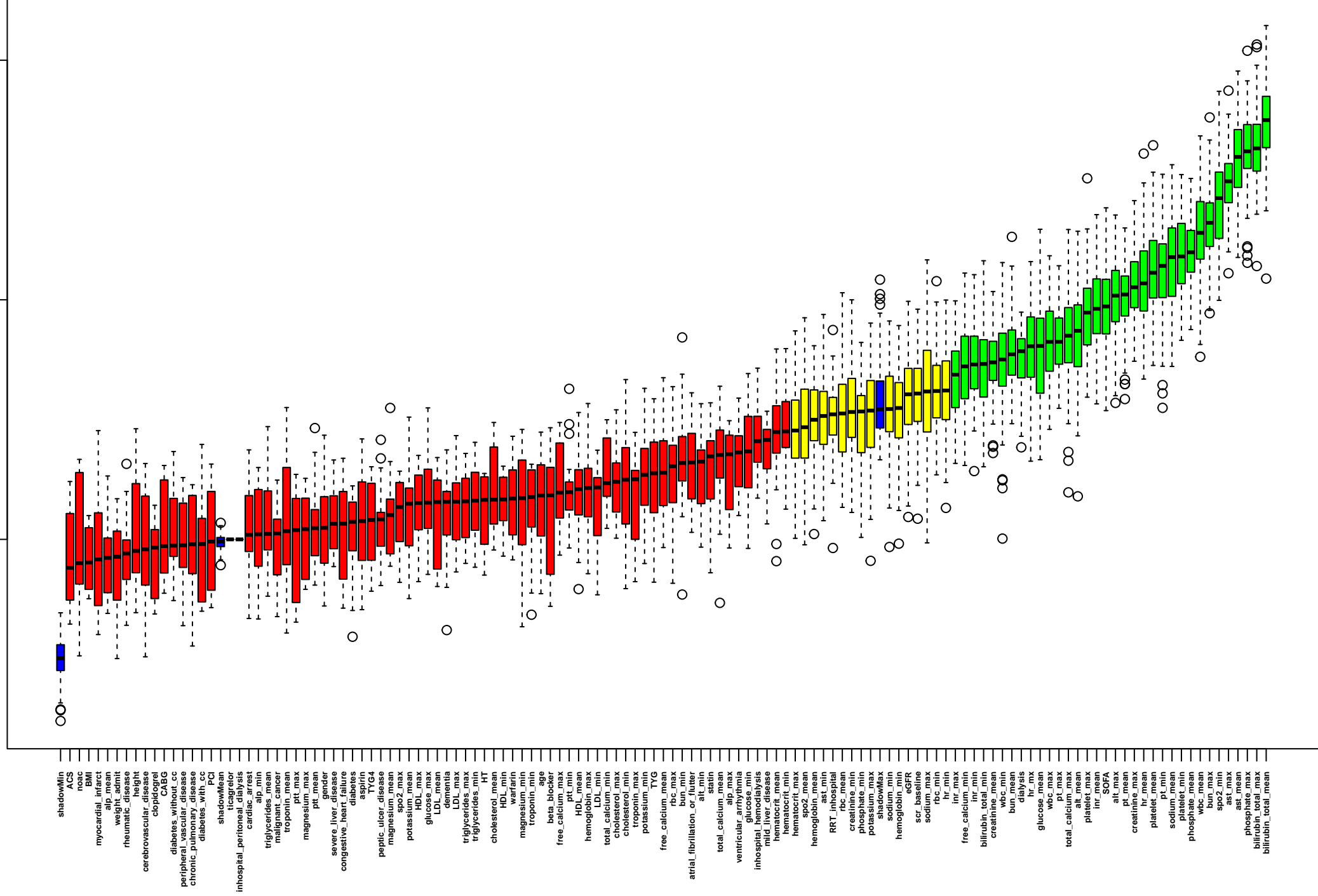

Supplement: Supplementary file 1 — Additional file 1: Figure S1 Feature selection for the relationship between various TyG indices and one-year mortality analyzed by the Boruta algorithm. [file 12933_2023_1843_MOESM1_ESM.pdf]
